# Functional limitation as a mediator of the relationship between multimorbidity on health-related quality of life in Australia: evidence from a national panel mediation analysis

**DOI:** 10.3389/fmed.2023.1151310

**Published:** 2023-05-17

**Authors:** John Tayu Lee, Marie Ishida, Tilahun Haregu, Sanghamitra Pati, Yang Zhao, Raffaele Palladino, Kanya Anindya, Rifat Atun, Brian Oldenburg, Tiara Marthias

**Affiliations:** ^1^Department of Primary Care and Public Health, Imperial College London, London, United Kingdom; ^2^Department of Global Health and Population, Harvard T.H. Chan School of Public Health, Boston, MA, United States; ^3^College of Health and Medicine, Australian National University, Canberra, ACT, Australia; ^4^Nossal Institute for Global Health, Melbourne School of Population and Global Health, The University of Melbourne, Parkville, VIC, Australia; ^5^ICMR Regional Medical Research Centre, Bhubaneswar, Odisha, India; ^6^The George Institute for Global Health China, Beijing, China; ^7^Department of Public Health, University “Federico II” of Naples, Naples, Italy; ^8^Interdepartmental Center for Research in Healthcare Management and Innovation in Healthcare (CIRMIS), University “Federico II” of Naples, Naples, Italy; ^9^School of Public Health and Community Medicine, Institution of Medicine, Sahlgrenska Academy, University of Gothenburg, Gothenburg, Sweden; ^10^Countway Library, Harvard Medical School, Boston, MA, United States; ^11^Implementation Science Lab, Baker Heart and Diabetes Institute, Melbourne, VIC, Australia; ^12^School of Psychology and Public Health, La Trobe University, Melbourne, VIC, Australia; ^13^Department of Health Policy and Management, Faculty of Medicine, Public Health and Nursing, Gadjah Mada University, Yogyakarta, Indonesia

**Keywords:** chronic disease, multimorbidity, mediation, functional limitation, comorbidity

## Abstract

**Objective:**

The inverse relationships between chronic disease multimorbidity and health-related quality of life (HRQoL) have been well-documented in the literature. However, the mechanism underlying this relationship remains largely unknown. This is the first study to look into the potential role of functional limitation as a mediator in the relationship between multimorbidity and HRQoL.

**Methods:**

This study utilized three recent waves of nationally representative longitudinal Household, Income, and Labor Dynamics in Australia (HILDA) surveys from 2009 to 2017 (*n* = 6,814). A panel mediation analysis was performed to assess the role of functional limitation as a mediator in the relationship between multimorbidity and HRQoL. The natural direct effect (NDE), indirect effect (NIE), marginal total effect (MTE), and percentage mediated were used to calculate the levels of the mediation effect.

**Results:**

This study found that functional limitation is a significant mediator in the relationship between multimorbidity and HRQoL. In the logistic regression analysis, the negative impact of multimorbidity on HRQoL was reduced after functional limitation was included in the regression model. In the panel mediation analysis, our results suggested that functional limitation mediated ~27.2% (*p* < 0.05) of the link between multimorbidity and the composite SF-36 score for HRQoL. Functional limitation also mediated the relationship between the number of chronic conditions and HRQoL for each of the eight SF-36 dimensions, with a proportion mediated ranging from 18.4 to 28.8% (*p* < 0.05).

**Conclusion:**

Functional status has a significant impact on HRQoL in multimorbid patients. Treatment should concentrate on interventions that improve patients' functioning and mitigate the negative effects of multimorbidity.

## Introduction

Australians are living longer lives than ever before, but many are dealing with multiple chronic diseases, resulting in a low health-related quality of life (1–3). With an aging population and increased risk factor exposure, the prevalence of multimorbidity, defined as the coexistence of two or more long-term conditions, is expected to rise further in Australia (4–7). Recent national studies in Australia found that the prevalence of non-communicable diseases (NCDs) was ~ 25% for those aged 50–54, and this percentage increased to 50% for those aged 70 and above, based on only 12 self-reported NCDs (8, 9). It is also worth noting that multimorbidity is highly socioeconomically patterned in Australia, as it is in many other high-income countries, with lower socioeconomic groups or aboriginal groups having a higher prevalence of multimorbidity (10). Multimorbidity imposes heavy costs on the patients, their families, the healthcare system, and society as a whole (11–14).

Health-related quality of life (HRQoL) is an important health outcome metric that assesses an individual's subjective physical and mental health. HRQoL, according to the WHO definition, is frequently multifaceted, encompassing both physical and mental health, and represents individuals' wellbeing in the context of their culture and values (15, 16). HRQoL is frequently assessed in clinical settings and health surveys to track patient wellbeing and progress toward national health goals (17–20). Poor HRQoL is associated with increased healthcare utilization, suboptimal treatment outcomes, and an increased risk of death (19, 21, 22).

Several studies, including high-level systematic reviews, have suggested that multimorbidity reduces HRQoL (23–25). A number of long-term health conditions were found to have a negative impact on HRQoL after controlling for confounding variables (23, 25). Numerous studies have discovered a link between functional limitation and poor HRQoL, with the risk of poor HRQoL increasing as functional limitation increases (26–28). However, it is unclear how much adjusting for functional limitation modifies or reduces the relationship between multimorbidity and HRQoL. Multimorbidity appears to be independently associated with poor HRQoL in the majority of studies reported to date, even after adjusting for functional limitation, though the magnitude of the association appears to be less significant when functional limitation is included in the models (23, 24, 28, 29). Similarly, multimorbidity does not fully explain the link between functional limitation and poor HRQoL.

Until now, the complex relationship between multimorbidity, functional function, and HRQoL has received insufficient attention. A mediation analysis is a statistical model that looks at the relationships between two variables and how much they are mediated by a third variable (e.g., the mediator). In the current epidemiological and public health literature, this methodology is widely used to assess the mechanism by which disease affects health outcomes. To address this important evidence gap in the literature, this is the first longitudinal study to investigate the role of functional limitation in the influence of multimorbidity on HRQoL using three waves of nationally representative data in Australia. This study looks specifically at the role of functional limitation as a mediator in the relationship between multimorbidity and HRQoL in a population-based sample of Australian adults.

## Methods

### Sample and data

This study utilized three waves of longitudinal data from Waves 9 (2009), 13 (2013), and 17 (2017) of the Household, Income, and Labor Dynamics in Australia (HILDA) survey. The HILDA survey is a nationally representative, household-based panel study that collects information on the health, wellbeing, socioeconomics, and labor market dynamics of Australian residents over the course of their lives. Commenced in 2001, data were collected annually through interviews with individuals aged 15 years and above from the selected households. Wave 1 (2001) data were collected from 13,969 individuals, and a sample top-up of 4,009 individuals was added in wave 11 (2011). A detailed description of the survey objectives and methods is provided elsewhere (30). In this study, respondents who participated in all three waves (Waves 9, 13, and 17) are included in the sample and dropped those with any missing data on covariates, leaving 6,814 respondents.

### HRQoL (outcome variable)

HILDA survey contains 36-item short questionnaires deriving from SF-36 Health Survey, which is a widely used instrument for assessing HRQoL. SF-36 includes eight dimensions of scale scores ranging from 0 to 100 (a higher score indicates better health) that measure physical and mental health: physical functioning (PF), role physical (RF), bodily pain (BP), general health (GH), vitality (VT), social functioning (SF), role emotional (RE), and mental health (MH). The overall health status of the individual was based on the overall SF-6D score, which is derived from the SF-36. Finally, these scores were converted to binary variables with a threshold of 25th percentile as 1 indicating poor health and 0 indicating better health.

### Multimorbidity (exposure variable)

The physical conditions included in HILDA were as follows: arthritis/osteoporosis, asthma, cancer, chronic bronchitis/emphysema, type 1 diabetes, type 2 diabetes, heart disease, high blood pressure/hypertension, and any other serious circulatory condition, depression/anxiety, and “other mental illnesses.” Respondents who answered affirmatively to the question “Have you been told by a doctor or nurse that you have any of these conditions?” were defined as reporting a health condition. We counted the number of self-reported health conditions to quantify the number of physical conditions (0, 1, 2, 3, 4, etc).

### Functional limitation (mediator)

The HILDA survey questionnaire inquired about respondents' functional limitations. Respondents were classified as having functional limitations if they answered yes to the following question: “Do you have any long-term condition, impairment or disability that restricts you in your everyday activities, and has lasted or is likely to last, for 6 months or more?.”

### Covariates

The covariates of this study are as follows: sex (men and women), age group (18–29, 30–39, and 40–49), marital status (married/cohabitating and single/separated/divorced/widowed), geographical region of Australia by state (New South Wales, Victoria, Queensland, Western Australia, South Australia, Tasmania, Northern Territory, and Australian Capital Territory), and locality (urban and rural). Socioeconomic status was obtained using the quintile of Socio-Economic Indexes for Areas (SEIFA) relative to socioeconomic advantage/disadvantage (SEIFA = 1: lowest SES group; SEIFA = 5: highest SES group).

### Statistical analysis

We adopted a panel mediation study design to explore the role of functional limitations (2013) on the relationships between multimorbidity and HRQoL using three waves of panel datasets. We performed the analysis following the steps suggested by Baron and Kenny, which consists of a series of panel data regression analyses (31). We examined the relationship between multimorbidity and HRQoL measures; the association between functional limitation and multimorbidity; and the association between HRQoL and multimorbidity and functional limitation. Based on the results, we identified the set of associations that meet the mediation criteria: (1) The multimorbidity is significantly associated with the functional limitation; (2) multimorbidity is significantly associated with HRQoL; (3) functional limitation is significantly associated with the HRQoL; and (4) the association between the multimorbidity and HRQoL is attenuated when the mediator is included in the model. To confirm the mediation effect, separate mediation models using the Stata paramed program (32) were adopted for each selected HRQoL measure, including each dimension of SF-36. The paramed command performs causal mediation analysis using parametric regression models and is based on a counterfactual approach. We further reported the estimated natural direct effect (DE), natural indirect effect (IE), and marginal total effect (TE) on the odds ratio scale. Percent mediation was estimated as the ratio of natural logarithms of the difference between TE and DE divided by TE. Furthermore, we examined the mediation effect of functional limitation across different dimensions of HRQoL outcomes by performing the same analysis on each HRQoL component. All estimates from mediation analysis were bootstrapped with 500 replications to obtain bias-corrected 95%CI.

All statistical analyses were performed using Stata 15 Corp. The panel data mediation analysis was conducted using the Stata paramed program, and a significance was set at 0.05. All statistical models were adjusted for the covariates mentioned above.

## Results

Table 1 presents the sample characteristics of this study. Of 6,814 participants, 53.5% were women, 71.9 were married/*de facto*, and 85.6% were residing in urban areas. In total, 55.2% reported not having any NCD, while 7.2% reported having three or more NCDs and 20.7% of the sample reported disability.

**Table 1:** Sample characteristics from wave 17 (year 2017).

		**Number of individual respondents (%)**
Total		6,814 (100)
Gender	Male	3,168 (46.5)
	Female	3,646 (53.5)
Age group	15–29	786 (11.5)
	30–49	2,337 (34.3)
	50–59	1,442 (21.2)
	60+	2,249 (33.0)
Marital status	Married/defacto	4,901 (71.9)
	Not married	1,913 (28.1)
Indigenous status	Non-Indigenous	6,708 (98.4)
	Indigenous	106 (1.6)
State	New South Wales	1,912 (28.1)
	Victoria	1,679 (24.6)
	Queensland	1,524 (22.4)
	South Australia	600 (8.8)
	Western Australia	672 (9.9)
	Tasmania	214 (3.1)
	Northern Territory	56 (0.8)
	Australian Capital Territory	157 (2.3)
Locality	Urban	5,831 (85.6)
	Rural	983 (14.4)
SEIFA	Highest	1,535 (22.5)
	Second highest	1,464 (21.5)
	Middle	1,352 (19.8)
	Second lowest	1,321 (19.4)
	Lowest	1,147 (16.8)
Number of chronic diseases	None	3,760 (55.2)
	1	1,747 (25.6)
	2	814 (12.0)
	3+	493 (7.2)
Functional limitation	No	5,405 (79.3)
	Yes	1,409 (20.7)

The participant's characteristics against key indicators are presented in Table 2. The mean number of NCDs was 0.74 for the overall sample. The number of NCDs and the prevalence of functional limitation were higher among the older age group and those with lower socioeconomic status. Finally, the proportion of low HRQoL showed a similar trend with functional limitation. The prevalence of low HRQoL was 24.8% among the overall sample. It was higher among people from the lower socioeconomic area (34.9%). However, women showed a higher prevalence of low HRQoL (26.8%), while men showed a higher prevalence of disability (21.6%).

**Table 2:** Sample characteristics against outcome variables from wave 17.

		**Mean number of NCDs**	**Prevalence of Disability (%)**	**Prevalence of Low HRQoL (%)**
Total		0.74	20.70	24.76
Gender	Male	0.72	21.60	22.35
	Female	0.77	19.90	26.84
Age group	15–29	0.16	8.14	19.85
	30–49	0.31	11.71	20.78
	50–59	0.70	17.82	23.93
	60+	1.44	36.15	31.13
Marital status	Married/defacto	0.71	19.10	21.06
	Not married	0.85	24.80	34.20
Indigenous status	Non-Indigenous	0.75	20.62	24.54
	Indigenous	0.73	24.53	38.68
State	New South Wales	0.78	21.53	23.93
	Victoria	0.69	17.69	25.13
	Queensland	0.75	21.11	25.18
	South Australia	0.87	23.96	26.79
	Western Australia	0.74	21.84	23.48
	Tasmania	0.87	26.17	30.84
	Northern territory	0.38	14.29	10.71
	Australian capital territory	0.55	14.65	21.02
Locality	Urban	0.74	20.10	25.11
	Rural	0.78	24.00	22.66
SEIFA	Highest	0.56	14.14	18.24
	Second highest	0.65	17.35	19.81
	Middle	0.74	20.71	25.00
	Second lowest	0.84	21.65	28.77
	Lowest	1.03	32.43	34.87

Table 3 shows the descriptive statistics of the HRQoL outcomes (overall HRQoL as well as the eight individual dimensions). The table also showed the univariate relationship among the number of NCDs, functional limitation, and HRQoL outcomes. The analysis found that having a higher number of NCDs was linked to poorer HRQoL outcomes, as demonstrated by the lower scores on the SF-6D (OR = 1.74, *p* < 0.05) and physical functioning (OR = 2.11, *p* < 0.05) scales. Additionally, the second equation revealed a significant correlation between the number of NCDs and functional limitation (OR: 1.94, *p* < 0.05). The third equation, which included both HRQoL measures and functional limitation in the model, revealed that the number of NCDs and functional limitation were both significantly associated with poor HRQoL outcomes. It is important to note that when functional limitation is included in the model, the magnitude of the effect between multimorbidity and HRQoL decreases in all measures, indicating that the impact of the number of NCDs on HRQoL outcomes was partially mediated by functional limitation.

**Table 3:** Mediation analysis using the method by Baron and Kenny.

	**Equation 1**		**Equation 2**		**Equation 3 (include both number of NCDs and functional limitation in the regression model)**			
	**Number of NCDs**		**Number of NCDs**		**Number of NCDs**		**Functional limitation**	
	**OR**	***p*** **-value**	**OR**	***p*** **-value**	**OR**	***p*** **-value**	**OR**	***p*** **-value**
Overall poor HRQoL	1.74	<0.001			1.52	<0.001	3.70	<0.001
**By component of HRQoL**								
Physical functioning	2.11	<0.001			1.84	<0.001	4.51	<0.001
Role physical	1.74	<0.001			1.51	<0.001	3.85	<0.001
Bodily pain	1.74	<0.001			1.52	<0.001	3.93	<0.001
General health	2.08	<0.001			1.86	<0.001	3.36	<0.001
Vitality	1.64	<0.001			1.47	<0.001	2.75	<0.001
Social function	1.69	<0.001			1.48	<0.001	3.29	<0.001
Role emotion	1.53	<0.001			1.38	<0.001	2.41	<0.001
Mental health	1.45	<0.001			1.32	<0.001	2.25	<0.001
Functional limitation			1.94	<0.001				

### Overall SF-6D score

**Figure 1:**
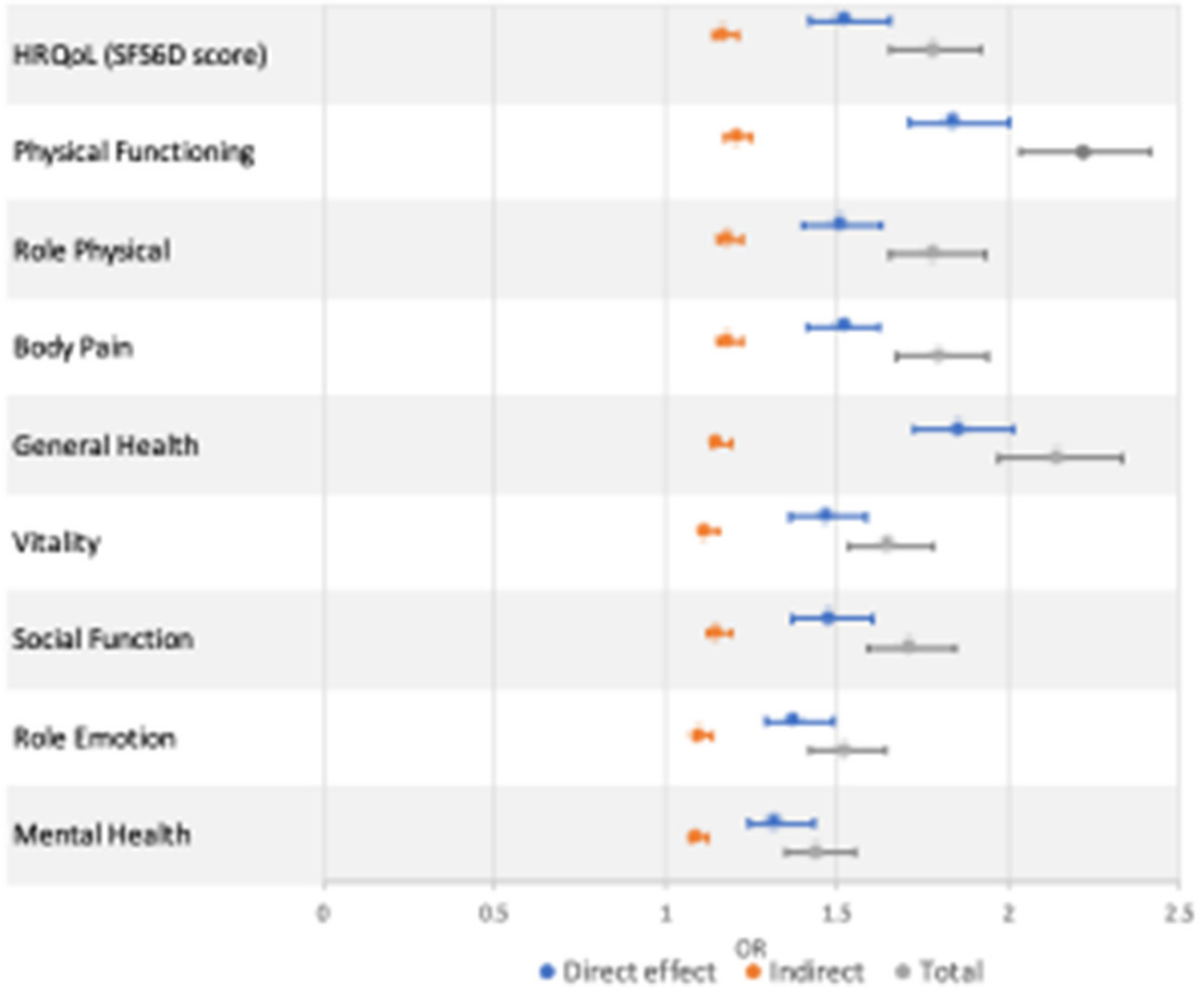
Direct, indirect, and total effect of multimorbidity on HRQoL.

Table 4 and Figure 1 show DE, IE, and TE of functional limitation on the overall SF-6D score and each dimension of SF-36. The number of NCDs was associated with lower overall HRQoL scores (OR^TE^:1.78, 95%CI: 1.65–1.92), with 27.2% mediated by functional limitation. The decomposition of total effects indicated statistically significant NDE (OR^DE^: 1.52, 95%CI: 1.42–1.65) and IE (OR^IE^: 1.17, 95%CI: 1.14–1.21).

**Table 4:** Results from panel mediation analysis.

	**Direct effect**		**Indirect effect**		**Total effect**		**% mediated by disability**
	**OR (95% CI)**	***p*** **-value**	**OR (95% CI)**	***p*** **-value**	**OR (95% CI)**	***p*** **-value**	
Overall HRQoL	1.52 (1.42, 1.65)	<0.01	1.17 (1.14, 1.21)	<0.01	1.78 (1.65, 1.92)	<0.01	27.4
**By components of HRQoL**							
Physical functioning	1.84 (1.71, 2.00)	<0.01	1.21 (1.17, 1.25)	<0.01	2.22 (2.03, 2.41)	<0.01	23.5
Role physical	1.51 (1.40, 1.63)	<0.01	1.18 (1.15, 1.22)	<0.01	1.78 (1.65, 1.93)	<0.01	28.5
Bodily pain	1.52 (1.41, 1.62)	<0.01	1.18 (1.15, 1.22)	<0.01	1.80 (1.67, 1.94)	<0.01	28.8
General health	1.86 (1.72, 2.01)	<0.01	1.15 (1.13, 1.19)	<0.01	2.14 (1.97, 2.33)	<0.01	18.4
Vitality	1.47 (1.36, 1.58)	<0.01	1.12 (1.10, 1.15)	<0.01	1.65 (1.53, 1.78)	<0.01	23.1
Social function	1.48 (1.37, 1.60)	<0.01	1.15 (1.12, 1.19)	<0.01	1.71 (1.59, 1.84)	<0.01	26.9
Role emotion	1.38 (1.29, 1.49)	<0.01	1.10 (1.08, 1.13)	<0.01	1.52 (1.42, 1.64)	<0.01	23.1
Mental health	1.32 (1.24, 1.43)	<0.01	1.09 (1.07, 1.12)	<0.01	1.44 (1.35, 1.55)	<0.01	23.9

### Each HRQoL dimensions

We also investigated the role of functional limitation in each dimension of the SF-36 separately. The most significant result was observed for physical functioning (OR^TE^: 2.22, 95%CI: 2.03–2.41) and general health (OR^TE^: 2.14, 95%CI: 1.97, 2.33), the associations of which were 23.9 and 18.4%, respectively, mediated by functional limitation. Functional limitation showed the largest mediation effect of the association between the number of NCDs and role physical (28.7%). Consistent results were found in other SF-36 domains (body pain: 28.2%, vitality: 22.6%, social function: 26.1%, role emotion: 22.8%, mental health: 23.6%).

## Discussion

### Principal findings and interpretation

This is the first study that uses a panel data study design to investigate the role of functional limitation as a mediator in the relationship between multimorbidity and health-related quality of life in Australia. We found clear evidence that functional limitation acts as a partial mediator on the relationships between multimorbidity and the overall HRQoL score as well as each SF-6D dimension, with the percentage of mediation ranging from 18 to 28%.

The possible explanation is that disabled older adults or those with greater functional limitations have a negative impact on their self-care ability, physiological activities, and social interaction, all of which have a negative impact on their physical and mental health, reducing HRQoL. Multimorbidity, functional limitation, and disability all have a negative impact on HRQoL outcomes (26, 28, 33, 34). Although it is widely assumed that multimorbidity is associated with poorer HRQoL, the mechanisms through which multimorbidity negatively affects HRQoL through functional limitation or disability have not been thoroughly investigated. Our findings show that functional limitation plays a role in mediating the relationship between multimorbidity and HRQoL.

### Clinical implications

Multimorbidity has been found to have a negative impact on the health-related quality of life of patients, partly due to its association with functional decline. Therefore, managing functional limitations effectively could help maintain the HRQoL of patients with multimorbidity. This implies a need for a more comprehensive approach to managing both conditions and recognizing their equal importance, which could ultimately improve the overall wellbeing of patients. In clinical practice, a thorough assessment of the various types of functional limitations in patients with multimorbidity could help guide appropriate preventive and supportive care, not only to maintain HRQoL but also to prevent further decline in function dependence.

## Limitations

This study has several limitations, including the use of self-reported measures which may be subject to recall and social desirability bias. Although a well-validated questionnaire was used to measure HRQoL, subjective measures may not fully capture the objective reality of patients' health status. Additionally, the study is not causal and only identified associations between variables. Although panel data was used, unobserved confounding variables cannot be completely ruled out. Notably, important clinical factors such as disease severity and duration of the illness were not included in the study, which could have affected the relationship between multimorbidity and HRQoL.

Furthermore, the mechanisms underlying the relationship between multimorbidity and HRQoL are complex and may involve multiple mediators. While the current study focused on function limitation as a mediator, other mediators such as psychological distress or social isolation may also play vital roles. Further research utilizing more comprehensive measures and sophisticated statistical techniques, such as structural equation modeling, may offer more insights into the mechanisms underlying the relationship between multimorbidity and HRQoL.

## Conclusion

This study contributes to the existing literature to understand the mediating effect of functional limitation and disability on the relationships between multimorbidity and HRQoL. Our study highlights the importance of improving patients' functional ability and preventing disability for patients with multimorbidity to improve their HRQoL. Therefore, healthcare that improves the management of patients with multimorbidity should include interventions that aim to encourage patients to participate in physical activity and increase social participation. Given the important mediating role, our study highlights that functional limitation or disability might be considered an intermediate outcome for evaluating interventions aimed at managing multimorbidity.

## Data availability statement

The study utilized HILDA survey in Australia, which can be apply for access to the dataset through the following link: https://melbourneinstitute.unimelb.edu.au/hilda.

## Author contributions

The aim of the research was developed by JL and MI. The methodology development and analysis were conducted by JL, MI, and TH. JL and MI drafted the manuscript draft. SP, YZ, RP, KA, RA, and BO contributed to all sections. All authors reviewed, edited, and commented on multiple versions of the manuscript. All authors contributed to the article and approved the submitted version.

## Figures and Tables

**Figure A1:**
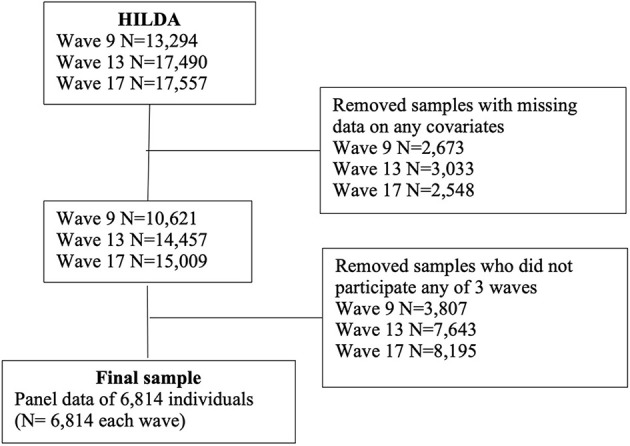
Sample flowchart.

## References

[1] Australian Institute of Health and Welfare. National Strategic Framework for Chronic Conditions, Reporting Framework: Indicator Results. (2022). Available online at: https://www.aihw.gov.au/getmedia/891c2a30-800b-4117-919f-fde48ef9f2f9/aihw-phe-299.pdf.aspx?inline=true (accessed January 26, 2023).

[2] Australian Institute of Health and Welfare. Chronic Condition Multimorbidity. Australian Institute of Health and Welfare (2021). Available online at: https://www.aihw.gov.au/reports/chronic-disease/chronic-condition-multimorbidity/contents/chronic-conditions-and-multimorbidity (accessed January 26, 2023).

[3] Australian Institute of Health and Welfare. Chronic Conditions and Multimorbidity. Australian Institute of Health and Welfare (2022). Available online at: https://www.aihw.gov.au/reports/australias-health/chronic-conditions-and-multimorbidity (accessed January 26, 2023).

[4] Palladino R Tayu Lee J Ashworth M Triassi M Millett C. Associations between multimorbidity, healthcare utilisation and health status: evidence from 16 European countries. Age Ageing. (2016) 45:431–5. 10.1093/ageing/afw044 27013499 PMC4846796

[5] Pan T Anindya K Devlin N Mercer SW McPake B van Heusden A. The impact of depression and physical multimorbidity on health-related quality of life in China: a national longitudinal quantile regression study. Sci Rep. (2022) 12:21620. 10.1038/s41598-022-25092-7 36517510 PMC9750988

[6] La DTV Zhao Y Arokiasamy P Atun R Mercer S Marthias T. Multimorbidity and out-of-pocket expenditure for medicines in China and India. BMJ Glob Health. (2022) 7:e007724. 10.1136/bmjgh-2021-007724 36328381 PMC9639066

[7] He L Biddle SJ Lee JT Duolikun N Zhang L Wang Z. The prevalence of multimorbidity and its association with physical activity and sleep duration in middle aged and elderly adults: a longitudinal analysis from China. Int J Behav Nutr Phys Activ. (2021) 18:1–12. 10.1186/s12966-021-01150-7 34112206 PMC8194125

[8] Sum G Salisbury C Koh GC-H Atun R Oldenburg B McPake B. Implications of multimorbidity patterns on health care utilisation and quality of life in middle-income countries: cross-sectional analysis. J Glob Health. (2019) 9:20413. 10.7189/jogh.09.020413 31448114 PMC6684869

[9] Ishida M Hulse ES Mahar RK Gunn J Atun R McPake B. The joint effect of physical multimorbidity and mental health conditions among adults in Australia. Prev Chronic Dis. (2020) 17:E157. 10.5888/pcd17.200155 33301391 PMC7769083

[10] Carman W Ishida M Trounson JS Mercer SW Anindya K Sum G. Epidemiology of physical–mental multimorbidity and its impact among Aboriginal and Torres Strait Islander in Australia: a cross-sectional analysis of a nationally representative sample. BMJ Open. (2022) 12:e054999. 10.1136/bmjopen-2021-054999 36220313 PMC9557280

[11] Lee JT Hamid F Pati S Atun R Millett C. Impact of noncommunicable disease multimorbidity on healthcare utilisation and out-of-pocket expenditures in middle-income countries: cross sectional analysis. PLoS ONE. (2015) 10:e0127199. 10.1371/journal.pone.0127199 26154083 PMC4496037

[12] Anindya K Ng N Atun R Marthias T Zhao Y McPake B. Effect of multimorbidity on utilisation and out-of-pocket expenditure in Indonesia: quantile regression analysis. BMC Health Serv Res. (2021) 21:427. 10.1186/s12913-021-06446-9 33952273 PMC8097787

[13] Pan T Mercer SW Zhao Y McPake B Desloge A Atun R. The association between mental-physical multimorbidity and disability, work productivity, and social participation in China: a panel data analysis. BMC Public Health. (2021) 21:376. 10.1186/s12889-021-10414-7 33602174 PMC7890601

[14] Zhao Y Atun R Oldenburg B McPake B Tang S Mercer SW. Physical multimorbidity, health service use, and catastrophic health expenditure by socioeconomic groups in China: an analysis of population-based panel data. Lancet Glob Health. (2020) 8:e840–9. 10.1016/S2214-109X(20)30127-3 32446349 PMC7241981

[15] Diener E Suh E. Measuring quality of life: economic, social, and subjective indicators. Soc Indic Res. (1997) 40:189–216. 10.1023/A:1006859511756

[16] World Health Organization. WHOQOL - Measuring Quality of Life. (2012). Available online at: https://www.who.int/tools/whoqol (accessed January 26, 2023).

[17] Götze H Taubenheim S Dietz A Lordick F Mehnert A. Comorbid conditions and health-related quality of life in long-term cancer survivors—associations with demographic and medical characteristics. J Cancer Survivorsh. (2018) 12:712–20. 10.1007/s11764-018-0708-6 30097854

[18] Peters M Potter CM Kelly L Fitzpatrick R. Self-efficacy and health-related quality of life: a cross-sectional study of primary care patients with multi-morbidity. Health Qual Life Outcomes. (2019) 17:37. 10.1186/s12955-019-1103-3 30764833 PMC6376655

[19] Lavados PM Hoffmeister L Moraga AM Vejar A Vidal C Gajardo C. Incidence, risk factors, prognosis, and health-related quality of life after stroke in a low-resource community in Chile (ÑANDU): a prospective population-based study. Lancet Glob Health. (2021) 9:e340–51. 10.1016/S2214-109X(20)30470-8 33422189

[20] Gaertner J Siemens W Meerpohl JJ Antes G Meffert C Xander C. Effect of specialist palliative care services on quality of life in adults with advanced incurable illness in hospital, hospice, or community settings: systematic review and meta-analysis. BMJ. (2017) 357:j2925. 10.1136/bmj.j2925 28676557 PMC5496011

[21] Zimbudzi E Lo C Ranasinha S Kerr PG Usherwood T Cass A. Self-management in patients with diabetes and chronic kidney disease is associated with incremental benefit in HRQOL. J Diabetes Complic. (2017) 31:427–32. 10.1016/j.jdiacomp.2016.10.027 27914731

[22] Van Wilder L Rammant E Clays E Devleesschauwer B Pauwels N De Smedt D. A comprehensive catalogue of EQ-5D scores in chronic disease: results of a systematic review. Qual Life Res. (2019) 28:3153–61. 10.1007/s11136-019-02300-y 31531840

[23] Fortin M Bravo G Hudon C Vanasse A Lapointe L. Prevalence of multimorbidity among adults seen in family practice. Ann Fam Med. (2005) 3:223–8. 10.1370/afm.272 15928225 PMC1466875

[24] Makovski TT Schmitz S Zeegers MP Stranges S van den Akker M. Multimorbidity and quality of life: systematic literature review and meta-analysis. Ageing Res Rev. (2019) 53:100903. 10.1016/j.arr.2019.04.005 31048032

[25] Pati S Swain S Knottnerus JA Metsemakers JF van den Akker M. Health related quality of life in multimorbidity: a primary-care based study from Odisha, India. Health Qual Life Outcomes. (2019) 17:116. 10.1186/s12955-019-1180-3 31277648 PMC6612103

[26] Eyowas FA Schneider M Balcha SA Pati S Getahun FA. Multimorbidity and health-related quality of life among patients attending chronic outpatient medical care in Bahir Dar, Northwest Ethiopia: the application of partial proportional odds model. PLoS Glob Public Health. (2022) 2:e0001176. 10.1371/journal.pgph.0001176 36962679 PMC10021695

[27] Wang Z Peng W Li M Li X Yang T Li C. Association between multimorbidity patterns and disability among older people covered by long-term care insurance in Shanghai, China. BMC Public Health. (2021) 21:418. 10.1186/s12889-021-10463-y 33639902 PMC7912511

[28] Williams JS Egede LE. The association between multimorbidity and quality of life, health status and functional disability. Am J Med Sci. (2016) 352:45–52. 10.1016/j.amjms.2016.03.004 27432034

[29] She R Yan Z Jiang H Vetrano DL Lau JTF Qiu C. Multimorbidity and health-related quality of life in old age: role of functional dependence and depressive symptoms. J Am Med Dir Assoc. (2019) 20:1143–9. 10.1016/j.jamda.2019.02.024 30979676

[30] Summerfield M Garrard B Hahn M Yihua J Roopa K Macalalad N. HILDA User Manual – Release 20. Melbourne, VIC: University of Melbourne (2021). Available online at: https://melbourneinstitute.unimelb.edu.au/__data/assets/pdf_file/0009/3969270/HILDA-User-Manual-Release-20.0.pdf (accessed March, 2023).

[31] Baron RM Kenny DA. The moderator–mediator variable distinction in social psychological research: conceptual, strategic, and statistical considerations. J Pers Soc Psychol. (1986) 51:1173. 10.1037//0022-3514.51.6.1173 3806354

[32] Emsley R Liu H. PARAMED: Stata Module to Perform Causal Mediation Analysis Using Parametric Regression Models, Statistical Software Components S457581. Boston College Department of Economics, Boston, MA, United States (2013). https://EconPapers.repec.org/RePEc:boc:bocode:s457581 (accessed January 23, 2023).

[33] Groessl EJ Kaplan RM Rejeski WJ Katula JA Glynn NW King AC. Physical activity and performance impact long-term quality of life in older adults at risk for major mobility disability. Am J Prevent Med. (2019) 56:141–6. 10.1016/j.amepre.2018.09.006 30573142 PMC6309909

[34] Wilk P Ruiz-Castell M Bohn T Fagherazzi G Nicholson K Moran V. Association between functional limitation and quality of life among older adults with multimorbidity in Luxembourg. Eur J Public Health. (2022) 32:ckac129-292. 10.1093/eurpub/ckac129.292

